# What Do Older People Think That Others Think of Them, and Does It Matter? The Role of Meta-Perceptions and Social Norms in the Prediction of Perceived Age Discrimination

**DOI:** 10.1037/pag0000125

**Published:** 2016-11

**Authors:** Christin-Melanie Vauclair, Maria Luísa Lima, Dominic Abrams, Hannah J. Swift, Christopher Bratt

**Affiliations:** 1 Centro de Investigação e Intervenção Social, Instituto Universitário de Lisboa; 2 Centre for the Study of Group Processes, School of Psychology, University of Kent

**Keywords:** age discrimination, older people, meta-perceptions, social norms, European Social Survey

## Abstract

Psychological theories of aging highlight the importance of social context. However, very little research has distinguished empirically between older people’s perception of how others in their social context perceive them (personal meta-perceptions) and the shared perceptions in society (societal meta-perceptions). Drawing on theories of intergroup relations and stereotyping and using a multilevel perspective, this article examines how well older people’s perceptions of age discrimination (PAD) are predicted by (a) older people’s personal meta-perceptions, (b) societal meta-perceptions, and (c) social norms of intolerance toward age prejudice. Aging meta-perceptions are differentiated into the cognitive and affective components of ageism. Multilevel analyses of data from the European Social Survey (*N*_over 70 years of age_ = 8,123, 29 countries; European Social Survey (ESS) Round 4 Data, 2008) confirmed that older people’s personal meta-perceptions of negative age stereotypes and specific intergroup emotions (pity, envy, contempt) are associated with higher PAD. However, at the societal-level, only paternalistic meta-perceptions were consistently associated with greater PAD. The results show that a few meta-perceptions operate only as a psychological phenomenon in explaining PAD, some carry consonant, and others carry contrasting effects at the societal-level of analysis. This evidence extends previous research on aging meta-perceptions by showing that both the content of meta-perceptions and the level of analysis at which they are assessed make distinct contributions to PAD. Moreover, social norms of intolerance of age prejudice have a larger statistical effect than societal meta-perceptions. Social interventions would benefit from considering these differential findings.

Individuals are embedded in a social context (Oishi & Graham, 2010), which is also considered to be crucial for understanding their aging experience (e.g., Levy, 2009). The present research uses evidence from the European Social Survey (ESS) to focus on two important social psychological components of the social context that should relate to older people’s experiences of age-discrimination, sometimes termed *perceived age discrimination* (PAD). These components are as follows: beliefs about how others generally perceive old age (referred to as meta-*perceptions*) and whether there is a normative climate that inhibits expressions of age prejudice. Aging perceptions can have powerful implications for older people: meta-analytic evidence shows that when negative aging perceptions are implied or explicit they can harm older individuals’ cognitive and physical functioning (Lamont, Swift, & Abrams, 2015) and health (e.g., Levy, 2009).

Only a couple of studies have examined how meta-perceptions may be related to older people’s PAD (Bowen & Staudinger, 2013; Garstka, Schmitt, Branscombe, & Hummert, 2004). Moreover, these studies with very small opportunity samples have focused only on specific meta-perceptions (e.g., meta-stereotypes) and have not distinguished between older people’s meta-perceptions and the perceptions that are actually widely shared in the social environment. Hence, it remains unknown how older people’s subjective views about how their age is perceived by others or how societal views about old age relate to experiences of age discrimination. Moreover, there are meta-perceptions of different elements of ageism (e.g., stereotypes and prejudices) and it is not known if these may have different relationships with older people’s PAD.

Social norms regarding the expression of ageism are a further aspect of the social context surrounding the expression and suppression of prejudice. These norms may also account for why individuals in some societies may experience more discrimination than in others (cf. Plant & Devine, 1998). Surprisingly little empirical research has examined the effects of normative contexts on individuals’ aging experience (e.g., Bowen & Staudinger, 2013).

In this article, we focus on these two understudied but highly relevant psychological topics: (a) drawing on theories of intergroup conflict and stereotyping we examine how meta-perceptions relate to older people’s PAD by taking a multilevel perspective (i.e., by studying older people’s personal meta-perceptions as well as societal meta-perceptions about old age as predictors), and (b) we assess whether antiprejudice norms create a social climate in which older people report less PAD.

## Meta-Perceptions

Meta-perceptions constitute a type of knowledge that is thought to be widely shared by others (Vorauer, Main, & O’Connell, 1998), and can be distinguished at the individual- and societal-levels. In this article, individual-level or personal meta-perceptions describe older people’s personal beliefs about how others in society perceive their age group. Research on meta-cognition and intergroup relations shows that individuals’ negative meta-perceptions may elicit heightened perceptions of discrimination (Frey & Tropp, 2006; Vorauer et al., 1998). Negative meta-perceptions inhibit intergroup contact by increasing anxiety about interactions with outgroup members (e.g., in simulated contact situations; Finchilescu, 2010) and increase potential for miscommunication and tension (Vorauer & Sakamoto, 2008). Individuals may infer from meta-perceptions whether they are liked by the outgroup and are likely to be targets of discrimination. Negative meta-perceptions are especially likely to be held by members of stigmatized groups (Pinel, 1999; Richeson & Shelton, 2006; Vorauer et al., 1998) as seems likely for older people. It is known that members of some stigmatized groups expect to be seen negatively by others and are more likely to perceive events as discriminatory (Pinel, 1999). This link has not been empirically tested yet or explored in relation to age despite the clear theoretical and empirical implication that older people’s personal meta-perceptions should be related to perceptions of age discrimination (PAD; Cuddy, Fiske, & Glick, 2007; Fiske, Cuddy, Glick, & Xu, 2002; Garstka et al., 2004).

Meta-perceptions are often assessed by asking respondents how a specific social group is viewed by most people in society (see, e.g., Fiske et al., 2002). Strictly speaking, these measures tap individual differences in meta-perceptions from which researchers may infer other individual differences in attitudes or behavior (cf. Chiu, Gelfand, Yamagishi, Shteynberg, & Wan, 2010). However, a higher order feature of meta-perceptions is the actual shared cultural perception which is represented by aggregating all individuals’ perceptions within the pertinent cultural context (Schwartz, 2013). These societal meta-perceptions represent societal views, reflecting the extent to which the majority in a cultural community shares a particular perception about a social group.

### Personal and Societal Meta-Perceptions

Empirical analyses on societal meta-perceptions have been limited to the study of media content (e.g., Donlon, Ashman, & Levy, 2005) or to small samples within a single country (for a review, see Kite, Stockdale, Whitley, & Johnson, 2005) or to cross-national student samples (Löckenhoff et al., 2009). Hence, the present research is the first to examine personal and societal meta-perceptions in large scale representative samples across multiple countries. We draw on the multilevel analysis literature (e.g., Bliese & Jex, 2002) to understand how personal and societal meta-perceptions may predict PAD.

In this article societal meta-perceptions represent the societal aging climate and are generated by aggregating individual (out) group members’ responses and calculating the group means. Our study examines cross-level direct-effect models (Bliese & Jex, 2002), that is, the link between meta-perceptions at the individual- and societal-level and PAD as an individual-level outcome variable. To do so, we will simultaneously regress older people’s PAD on both personal and societal meta-perceptions. If societal meta-perceptions are significantly related to PAD even when older people’s personal meta-perceptions are also in the model, this will demonstrate that there are distinguishable implications of the two levels of meta-perceptions. It is important to note that it would support the idea that societal meta-perceptions have emergent properties.

### Theoretical Considerations

The benefit of simultaneously modeling direct effects of individual- and societal-level constructs lies in the integration of different theoretical conceptualizations. The social representation perspective (Deaux & Philogène, 2001; Moscovici, 1988) suggests that societal level meta-perceptions influence PAD because, for example, if there is a social consensus that older people are incompetent, then older people should perceive age discrimination. Hence, it is an objective conceptualization of aging beliefs in which a certain societal aging climate is seen as universally negative for individuals’ psychological aging experience. Alternately, the meta-cognition perspective (e.g., Frey & Tropp, 2006; Vorauer et al., 1998) is a subjective conceptualization of aging beliefs by suggesting that negative meta-perceptions about aging relate to PAD, but only if they exist as personal meta-perceptions. Although both perspectives suggest that negative meta-perceptions about aging should be linked to PAD, they address different conceptualizations, and therefore, different levels of analyses. Thus far they have been considered separate in research; whereas in this article we consider that they both may have an influence in explaining PAD.

When meta-perceptions are examined at the individual- and societal-level, there is the possibility that they can produce congruent or distinctive influences on PAD. This is because the two levels of analyses are statistically independent (Bliese & Jex, 2002; see also Fischer, Vauclair, Fontaine, & Schwartz, 2010) and, therefore, the individual- and group-levels of meta-perceptions can exert independent effects on the criterion variable. The extent to which individual-level and societal-level meta-perceptions exert similar effects is known as isomorphism. We believe that there are good reasons to expect isomorphism to occur given that social representations of stereotypic beliefs (Deaux & Philogène, 2001; Moscovici, 1988) can have the same impact as personally endorsed stereotypic beliefs. There are two ways in which social consensus about aging beliefs may increase an older person’s vulnerability to age discrimination. First, older people may inadvertently display stereotype-confirming behavior, which can then lead others to behave toward them in ways that further reinforce the stereotype. Second, if a false consensus exists (, e.g., regarding older people’s competence), this is likely to be expressed through conversation, media imagery and in people’s assumptions. It therefore represents an unavoidable ‘threat in the air’ for older people, as posited by stereotype threat theory (Steele, 1997). Stereotype threat can also result in stereotype-confirming behavior such as substantial decrements in cognitive and physical performance (Lamont et al., 2015). This threat then creates a lens through which older people may perceive that they are not accorded the respect and treatment they deserve. As such, societal and personal meta-perceptions should exhibit very similar, or isomorphic, associations with PAD.

#### Elements of aging meta-perceptions

For our purposes, we adopt a tripartite model to conceptualize the content of meta-perceptions and age discrimination as part of the ageism syndrome, delineating different types of aging perceptions that have been flagged requiring closer attention in the literature (see Levy, 2009). *Ageism* can be defined as negative or positive stereotypes, prejudice, and/or discrimination against (or to the advantage of) elderly people on the basis of their chronological age or on the basis of a perception of them as being old (Iversen, Larsen, & Solem, 2009). On the basis of social psychological theories, we propose specific hypotheses about how different elements of aging meta-perceptions, tapping into stereotyping and prejudice, should relate to perceptions of discrimination, see Table 1.^1^

##### Perceived social status

Compared with middle-aged adults, older adults are commonly perceived as having lower social status in the sense of power, wealth, respect, influence, and prestige (Garstka et al., 2004). These perceptions affect the way older people are treated in social interactions (Fiske et al., 2002), and older adults are likely to attribute discriminatory practices to their membership in a low status age group (Garstka et al., 2004). Hence, older individuals who believe that their age group is seen as of low social status should also be more sensitive to discriminatory treatment because of age. Moreover, older people are attributed a much higher social status in some countries than in others (Vauclair et al., 2015b), which should have an effect on how older people feel they are treated in these societies.

*Hypothesis 1 (H1):* Meta-perceptions of the perceived social status of older people should predict older adult’s PAD negatively at the individual-level (H1a) and societal-level (H1b).

##### Age stereotyping

The stereotype content model suggests that two underlying dimensions, competence and warmth, organize the stereotypical perceptions associated with any social group in society (see Fiske et al., 2002). Ascribed competence is the degree to which a social group is characterized as intelligent and capable and ascribed warmth is the degree to which the social group is regarded as friendly and likable. A negative evaluation of a social group along these two dimensions should be related to discriminatory acts against this group (Cuddy et al., 2007) and therefore to the experience of discrimination. North and Fiske’s (2015) meta-analysis indicated that age stereotypes of competence and warmth evaluations do differ depending on the cultural context.

Hence, we expected the following:

*Hypothesis 2 (H2):* Meta-perceptions of warmth predict PAD negatively at the individual-level (H2a) and societal-level (H2b).

*Hypothesis 3 (H3):* Meta-perceptions of competence predict PAD negatively at the individual-level (H3a) and societal-level (H3b).^2^

##### Paternalistic prejudice

Previous research has shown that older people are usually pitied (Cuddy et al., 2007). Cuddy and colleagues (2007) suggested in their model on intergroup affect and behaviors that this ambivalent feeling should elicit discriminating behaviors (e.g., disrespect and neglect; Cuddy et al., 2007). Hence, PAD should be higher if older people think their age group is perceived in a paternalistic way and in societies in which they are commonly seen in a patronizing way.

*Hypothesis 4 (H4):* Meta-perceptions of pity felt toward older people predict older adult’s PAD positively at the individual-level (H4a) and societal-level (H4b).

##### Envious prejudice

For some individuals and societies, older people may be perceived as successfully trying to obtain more control over scarce resources, such as wealth and employment, and are therefore seen as disliked and untrustworthy (North & Fiske, 2012). According to the stereotype content model (Fiske et al., 2002), these feelings of envy should result in discriminatory behaviors (e.g., exclusion and rejection; see Cuddy et al., 2007). Hence, older people who think that others perceive them in this way may also report more PAD. Furthermore, older people in societies in which the predominant perception about old age is associated with an envious prejudice should also report more experiences with ageist behavior.

*Hypothesis 5 (H5):* Meta-perceptions of envy toward older people predict older adult’s PAD positively at the individual-level (H5a) and societal-level (H5b).

##### Contemptuous prejudice

Older people may also be the target of contemptuous prejudice (like other perceived societal free riders such as welfare recipients, see Fiske et al., 2002) if they are seen as a burden to society who contribute less than they benefit. Contemptuous prejudice elicits harmful behaviors such as exclusion and demeaning paternalistic behaviors (see Cuddy et al., 2007). Hence, older people who think that their age group is met with a contemptuous prejudice and those who live in societies that perceive senior citizens in this way should report more age discrimination.

*Hypothesis 6 (H6):* Meta-perceptions of contempt felt toward older people predict older adult’s PAD positively at the individual-level (H6a) and societal-level (H6b).

#### Social norms of intolerance of age prejudice

Some individuals are more motivated than others to avoid being prejudiced (Plant & Devine, 1998), and the same may be true of societies (in terms of the majority perspective) whose norms surrounding prejudice and the expression of prejudice can differ. Despite much social psychological research on this construct (see Kunstman, Plant, Zielaskowski, & LaCosse, 2013), to the best of our knowledge the motivation to be unprejudiced has not been studied to date as a form of social climate and as a societal-level predictor of social minority’s experience with discrimination.

We propose that prejudice norms are likely to create a social climate in which social minorities feel more or less accepted and that this should also be related to their perceptions of discrimination. A widely shared norm not to express prejudice could be the result of legislative changes protecting the rights of older people, or could stem from widely agreed upon politically correct standards, mandates for proper speech and behavior (e.g., O’Cinneide, 2005), as well as cultural values (cf. Vauclair & Fischer, 2011), which altogether create an external social pressure in society to value tolerance and avoid prejudice.

*Hypothesis 7 (H7):* The motivation to be age unprejudiced in society—representing a social norm—predicts older adult’s PAD negatively.

### Method

#### Data source

We used data from the European Social Survey (ESS) from Round 4 (European Social Survey Round 4 Data, 2008), collected through computer-based personal interviews in 29 countries from the European region, plus Israel, from 2008 to 2010. The survey uses random probability samples representative of the eligible residential populations in each country (aged 15 years and over). The ESS used a multistage pretesting and piloting process to ensure functional equivalence of all items at both conceptual and linguistic level. We used a subsample of older adults who are 70 years of age and beyond (*N* = 8,123; *M*_*age*_ = 76.87, *SD*_age_ = 5.43; 59.7% female) to study the link between older people’s personal meta-perceptions and PAD. Descriptive statistics for each country are in Table 2.[Table-anchor tbl2]

Items in the Experiences and Expressions of Ageism Module of the ESS use the age of over 70 to refer to older adults, reflecting both that it is beyond typical retirement ages for most countries and accommodates cross-country consensus that respondents in all countries regard this to be beyond the start of old age (see Abrams, Vauclair, & Swift, 2011). For the present analyses, the sample selection therefore ensures a direct correspondence between meta-perceptions targeting this age group and the PAD of this target age group.

#### Individual-level variables

##### Criterion variable

PAD was measured by the three questions, “How often, in the last year, has anyone shown prejudice against you or treated you unfairly because of your age?”; “And how often, if at all, in the last year have you felt that someone showed you a lack of respect because of your age, for instance by ignoring or patronizing you?; “How often in the last year has someone treated you badly because of your age, for example by insulting you, abusing you or refusing you services?” All response scales ranged from 0 = *never* to 4 = *very often*. Cronbach’s alpha was satisfactory for all countries ranging from .75 (Norway) to .94 (Ireland). Moreover, recent analyses showed that the data support approximate measurement invariance (approximately invariant factor loadings and approximately invariant intercepts) across the 29 countries (Bratt, Vauclair, Abrams, Swift, & Marques, 2016). Hence, we computed an overall index of PAD by averaging the scores on these three variables. The proportion of older people who scored above 1 on this index (i.e., who experienced age discrimination once or more often in the past year) ranges from 24% (Sweden) to 76% (Czech Republic) with a grand mean of 43% (see Table 2).

##### Covariates

Van den Heuvel and van Santvoort (2011) conducted a single-level analysis on individual-level predictors of older people’s PAD using ESS data. Because the following variables emerged as significant predictors we included them as covariates in the model: age, gender (1 = male, 2 = female), years of full-time education completed, subjective poverty (1 = *living comfortably on present income* to 4 = *finding it very difficult on present income*), born in country (1 = *yes*, 2 = *no*), perceived seriousness of age discrimination in general in respondents’ country (1 = *very serious* to 4 = *not at all serious*; recoded so that higher scores indicate greater perceived seriousness), life satisfaction (0 = *extremely dissatisfied* to 10 = *extremely satisfied*) and self-rated mental and physical health (1 = *very good* to 5 = *very bad*).^3^ Generalized trust was assessed through three questions measuring trust in others (0 = *you can’t be too careful* to 10 = *most people can be trusted*), perceived fairness (0 = *most people try to take advantage of me* to 10 = *most people try to be fair*), and perceived helpfulness (0 = *people mostly look out for themselves* to 10 = *people mostly try to be helpful*). The three items showed satisfactory internal reliability in each country (Cronbach’s alpha ranging from .60 in France to .88 in Cyprus) and were therefore averaged to an overall index of generalized trust.

##### Meta-perceptions

Status perceptions were measured with the question “Where would most people place the status of people over 70?” (0 = *extremely low status* to 10 = *extremely high status*). Status was defined for the respondents as referring to prestige, social standing or position in society.

Meta-perceptions of the warmth and competence dimension of age stereotypes were assessed with the two questions “How likely is it that most people in [country] view those over 70 . . . as friendly? . . . as competent?” (0 = *not at all likely to be viewed that way* to 4 = *very likely to be viewed that way*). The two items correlated significantly at the individual-level (*r* = .49, *p* < .001). Hence, we used a composite score (labeled *positive age stereotypes*) for analyses that involve both individual-level meta-stereotypes (see Table 4).[Table-anchor tbl3][Table-anchor tbl4]

The perceived intergroup emotions *pity*, *envy*, and *contempt* were measured with the three questions “How likely is it that most people in [country] view those over 70 . . . with pity? . . . with envy? . . . with contempt? The response scales ranged from 0 = *not at all likely to be viewed that way* to 4 = *very likely to be viewed that way*.^4^

All of these items have been tested in previous work in regard to their psychometric properties (see Vauclair, Abrams, & Bratt, 2010).

#### Country-level variables

##### Covariates

Taking into account previous findings, we also examined socioeconomic development (van den Heuvel & van Santvoort, 2011) and income inequality (Vauclair et al., 2015a) as country-level predictors of PAD. We used the Human Development Index (HDI) for the year 2007 (Human Development Report, 2009) as a proxy for the former. As a measure of income inequality in countries, we used the Gini coefficient from Eurostat for the year 2008.^5^ The Gini coefficient used for Turkey was only available for the year 2006. We complemented missing data in Eurostat with Gini coefficients from the World Income Inequality Database for Israel (from 2001), Russia (from 2006) and Ukraine (from 2006).^6^

##### Societal meta-perceptions

To assess the shared meta-perceptions about old age in society, we computed aggregated country-level scores from the ESS based on responses from individuals aged 69 years or under (*N* = 48,421, *M*_*age*_ = 42.62, *SD*_age_ = 15.05, 53.7% female; see also Table 2) for each of the following variables: perceived status, competence and warmth, pity, envy, and contempt. Note that contrary to the individual-level, the warmth and competence stereotype did not correlate significantly at the country-level (*r* = .03, *p* = .86). Hence, they were treated as separate country-level predictors in the full multilevel model (see Table 4).

##### Social norms

We used responses from individuals under 69 years of age on two items that assessed the internal and external motivation to respond without prejudice (Plant & Devine, 1998): “How important is it for you to be unprejudiced against people of other age groups” and “How important is it for you to be seen as being unprejudiced against people of other age groups” (0 = *not at all important* to 10 = *extremely important*). These two measures correlated very highly at the country-level, *r*(28) = .91, *p* < .001, so that we averaged them in order to obtain a composite score representing the shared social norm of being unprejudiced on the basis of age.

#### Statistical analysis

Given the clustered data structure (individuals nested within countries), we used multilevel regression analyses (with HLM 7.01, Raudenbush & Bryk, 2002) and tested the various effects of individual- and societal-level predictors on older people’s PAD. This enabled us to establish the extent to which older people’s personal meta-perceptions about their age group or the societal context in which they reside account for their PAD.

Our modeling strategy consisted of two main steps: First, we tested each personal and societal meta-perception separately as predictors of older people’s PAD in order to verify our hypotheses. We used group-mean centering for individual-level predictors as this is the most appropriate centering strategy when associations between predictors and outcome variable at the lowest level are of substantive interest (Enders & Tofighi, 2007). Next, we examined the predictor’s influence at the two levels by including the individual-level predictor together with its societal-level counterpart into the same model. We used grand-mean centering for all predictors here because any individual-level predictor under grand-mean centering is a composite of within- and between-cluster variation and can therefore be correlated with a Level 2 predictor. Most important, this means that if the magnitude of the association between the predictor and criterion variable is identical at both levels, the Level 2 predictor provides no additional explanatory power (Enders & Tofighi, 2007). If a societal meta-perception explains unique variance PAD over and above older people’s personal meta-perception, it provides evidence that the meta-perception has emergent properties when aggregated to the group level.

Second, we tested the full multilevel regression model in a stepwise procedure. We started with Model 0, which is the model without any predictors that reveals how much of the total variance in PAD is associated with country differences as opposed to individual differences (i.e., the intraclass correlation coefficient [ICC]). For Model 1a, we added individual-level covariates with only the intercepts varying randomly. In Model 1b, we examined the predictive power of individual-level meta-perceptions over and above individual-level covariates. Model 2a examines country-level covariates and Model 2b the social norm of intolerance of age prejudice as predictor of older people’s PAD. In the final model (Model 3) only country-level variables that emerged as significant predictors in previous analyses (see Table 3) are added. This strategy allowed us to preserve a maximum degree of freedom at the country-level. Models 2a, 2b, and 3 include individual-level covariates because this adjusts for possible compositional differences between countries in regard to variables that might also explain between-country differences in older people’s PAD. For example, previous research found that subjective ill-health is related to age discrimination (van den Heuvel & van Santvoort, 2011). By taking into account that some countries may be composed of a larger proportion of older people reporting ill-health than in other countries, we can assess the unique predictive power of meta-perceptions on the criterion variable.

### Results

#### Meta-perceptions at the individual- and societal-level

Table 3 summarizes the predictive relationships of meta-perceptions, regarding each element of ageism, on PAD at the different levels of analysis.

##### Status

Consistent with H1a, older adults with personal meta-perceptions of lower status of older people reported experiencing more age discrimination (higher PAD). Consistent with H1b, those living in *countries* in which the status of older people (societal meta-perception) was perceived to be lower also experienced more age discrimination. The predictive contribution of societal-level meta-perceptions was diminished but remained significant when controlling for older people’s personal meta-perceptions (*p* < .05).

##### Stereotypes

Consistent with H2a, respondents with meta-perceptions of older people as being more friendly also reported less PAD. Similarly, H2b was supported as higher societal meta-perceptions were also negatively related to PAD. However, including both levels of meta-perceptions in the model diminished the country-level effect indicating that the association between the two variables is the same across levels of analysis and that there is no explanatory gain in including the societal perceptions (cf. Bliese & Jex, 2002).

Older people’s personal meta-perceptions of competence were also significantly negatively related to PAD, consistent with H3a. However, societal meta-perceptions did not relate to PAD, ruling out H3b and indicating that this variable only related to PAD at the individual-level.

##### Emotions

In line with H4a and H4b, personal and societal meta-perceptions of pity related to PAD positively, and both remained significant predictors when they were included in the model, showing that pity meta-perceptions relate to discrimination simultaneously at both the individual- and societal-levels.^7^

In line with H5a, older people’s personal meta-perception of envy predicted PAD positively. Surprisingly, societal meta-perceptions were significantly negatively related to PAD (contrasting with H5b), indicating that opposing implications follow from individual- and societal meta-perceptions of envy toward older people.

Consistent with H6a, older people’s personal meta-perceptions of contempt were positively related to PAD, however there was no association between societal meta-perceptions of contempt and PAD (failing to support H6b). Hence, this variable operated at the individual-level only.

In sum, nine of the 12 hypotheses on meta-perceptions were confirmed. Among the disconfirmations, two were rejected at the societal-level (contempt and competence) and one result revealed a societal-level association that was opposite to what we found at the individual-level (envy).

#### Full multilevel regression models

Table 4 shows the full multilevel regression models in which we probed into the robustness of our findings in a stepwise procedure. Model 0 yielded an estimated grand mean of 0.53 (*SE* = 0.05, *p* < .001), indicating that on average across all ESS countries, older people experienced levels of age discrimination that were significantly above zero. Note that the grand-mean refers to incidents of age discrimination within the last year. Hence, responses are likely to be an underestimation when it comes to overall PAD.

The ICC showed that a large proportion of the total variance in older people’s PAD was due to individual differences (91.37%), and the remaining 8.63% of variance was associated with differences between countries. The chi-square statistic indicated that the differences in mean scores were significant, χ^*2*^(28) = 785.01, *p* < .001, justifying follow-up analyses using country-level predictors.

Model 1a shows the effects of covariates that have previously been related to PAD. Consistent with previous findings (van den Heuvel & van Santvoort, 2011), respondents’ age and whether they were born in the country were not significant predictors of experienced age discrimination. Respondents reported significantly more incidents of age discrimination if they perceived themselves to be poorer, less satisfied with their lives, reported greater subjective ill-health, had less trust in other people, and thought that age discrimination was a rather serious issue in society. Contrary to van den Heuvel and van Santvoort’s (2011) findings, which were based on ordinary regression analyses of the clustered ESS data, our multilevel results indicated that gender and level of education were not significant predictors of PAD. This set of individual-level predictors explained 8% of the within-country variance.

In Model 1b, we also tested all personal meta-perceptions as predictors of age discrimination. Note that friendliness and competence were here included as a composite score representing positive age stereotypes since the effect of competence was diminished when entered together with friendliness which is due to their relatively high individual-level correlation. This issue did not occur for all other meta-perceptions. Table 4 shows that the results remain unchanged compared to those reported in Table 3: PAD was lower among older respondents who perceived the status of their age group to be higher in society, who thought that they were stereotyped positively and met with little pity, envy and contempt. The model explained 17% of the variance in PAD and was significantly better than Model 1a, χ^2^(5) = 2181, *p* < .001.

In Model 2a we examined the socioeconomic context as country-level predictors of PAD after taking into account all individual-level predictors. We found no evidence that the HDI or income inequality were predictive of the criterion variable over and above the individual-level covariates. Hence, they were dropped from subsequent models.

Next, we found confirmation for H7 that the social norm of being non-age prejudiced should be significantly negatively related to older people’s PAD (Model 2b). In the final model, we added all societal meta-perceptions that had emerged as significant predictors in previous analyses as shown in Table 3. We verified that there was no issue of multicollinearity among the country-level predictors (*r*_maximum_ = .79). Model 3 shows that the social norm was the only variable that remained significant. Although Model 3 explained 81% of the between-country variance (compared with 75% in Model 2b), a chi-square test for nested models showed that there was no significant difference between Model 3 and Model 2b, χ^2^(4) = 8, *ns*. Hence, to understand why there are differences between countries in older people’s PAD, it is crucial to know whether there is a social norm of being unprejudiced toward other age groups or not. In societies in which there is a stronger shared norm of intolerance of age prejudice, older people do indeed report experiencing significantly less age discrimination.

**Figure 1 fig1:**
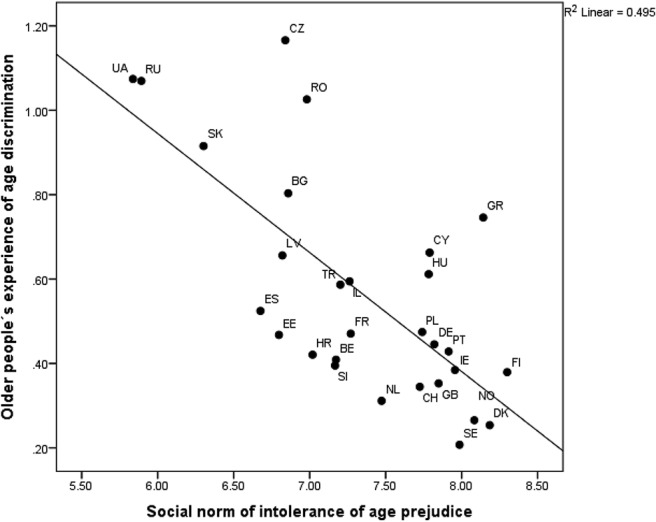
Association between frequency of perceived age discrimination scores for older people (above 70 years of age) in ESS countries (Belgium [BE], Bulgaria [BG], Switzerland [CH], Cyprus [CY], Czech Republic [CZ], Germany [DE], Denmark [DK], Estonia [EE], Spain [ES], Finland [FI], France [FR], United Kingdom [GB], Greece [GR], Croatia [HR], Hungary [HU], Israel [IL], Latvia [LV], Netherlands [NL], Norway [NO], Poland [PL], Portugal [PT], Romania [RO], Russian Federation [RU], Sweden [SE], Slovenia [SI], Slovakia [SK], Turkey [TR], Ukraine [UA]) and the social norm of intolerance of age prejudice.

Figure 1 shows the relationship between this country-level predictor and PAD scores across ESS countries. At the lower end of the slope, there is no coherent underlying pattern that can be identified. Some Nordic (e.g., Sweden, Norway), Western (e.g., Germany, Netherlands), Southern (e.g., Portugal, Cyprus), and Eastern European (e.g., Poland, Estonia) countries cluster here together. However, those that are located at the higher end of the slope tend to be mostly Eastern European countries (e.g., Ukraine, Slovakia).[Fig-anchor fig1]

### Discussion

#### Older people’s personal meta-perceptions

Consistent with previous research (Fiske et al., 2002; Garstka et al., 2004; Pinel, 1999, we found that if older people personally believe that their age group is seen as of lower social status and that others feel pity, contempt and envy toward them and stereotype them negatively, they also report experiencing more age discrimination. The fact that different types of emotions, which to some extent could be considered as incompatible (e.g., envy and contempt), were predictive of age discrimination suggest that there may be different subgroups of older people. Although the perception of subgroups of older people has been studied before (e.g., Brewer, Dull, & Lui, 1981; Schmidt & Boland, 1986), the present cross-national findings go well beyond the relatively limited samples from the American context in which they have been examined previously. Our study points to the possibility that older people may perceive and possibly identify with different old age subgroups that are not characterized by age boundaries (e.g., the young−old, old−old distinction), but by sociopsychological characteristics (e.g., the pitied seniors with low social status vs. the envied seniors with high social status). This opens up new research avenues in regard to self-categorization and identification with these groups and their implications for successful aging. Considering sociopsychological diversity in old age would also reconcile the different ageism theories which have been proposed in the literature (for a review, see North & Fiske, 2012).

#### Societal meta-perceptions

Societal meta-perceptions regarding older people’s status and the specific emotions of pity and envy felt toward them were predictive of older people’s PAD after controlling for the individual-level counterparts. The status and pity results are consistent with our hypotheses (see also, Cuddy et al., 2007; Fiske et al., 2002), but the finding that envy relates negatively to older people’s PAD, and therefore shows a different sign than at the individual-level, is surprising. This nonisomorphism across levels of analyses points to different meanings of envy at the individual- and societal-level (cf. Bliese & Jex, 2002). It seems that there are ageing meta-perceptions that vary from the default perception of pity (cf. North & Fiske, 2012). It appears that there is a specific image of (a subgroup of) older people in society that is envied. For instance, the baby boomer generation is usually portrayed as retiring with good health, having a relatively high education, solid financial resources and as enjoying a comfortable lifestyle. This may lead to a shared perception of older people and retirement which elicits envy, but of a benign type which involves the motivation to improve one’s own position and to achieve the same goals—as opposed to a malicious type, which is the motivation to cause damage to the target (see Abrams & Vasiljevic, 2014; van de Ven, Zeelenberg, & Pieters, 2009). In a societal context in which older people are seen this way, they may not encounter much age discrimination. On the other hand, older adults who personally believe that others envy their age group may be especially sensitive to any discriminatory practice that they interpret as an expression of a malicious form of envy. This discrepancy in perceptions and its contrasting implications for older people’s aging experience is worth exploring in future research into approaches for improving intergenerational relations, especially from the perspective of older people.

#### Individual- versus societal-level associations

The predictive power of societal meta-perceptions of friendliness and social status was diminished considerably when controlling for the individual-level counterparts. This indicates that they carry similar meaning at both levels of analysis. Thus, knowing about the societal views for these elements does not make any significant contribution in understanding older people’s PAD (cf. Bliese & Jex, 2002). This may be because of the shared knowledge that a concomitant of old age is the retirement from professional activities which diminishes the perceived social status of older people (see also Vauclair et al., 2015b). The result referring to friendliness may have evolutionary underpinnings and, therefore, also the same meaning at the individual- and societal-level. Evolutionary theories suggest that older people’s physical (e.g., baldness in men, softer voices) and specific intellectual attributes (e.g., wisdom) signal warmth and friendliness which convey nonthreatening characteristics. They may have been selected for in the course of evolution because they inhibit attack by other group members and therefore preserve older people’s valuable knowledge and experience (see Zebrowitz & Montepare, 2002).

Especially intriguing are those aging perceptions that show non-isomorphic effects across levels of analyses. They point to distinctive and sometimes contrasting effects on experiences of discrimination associated with older people’s personal views and those endorsed by society. Whereas envy perceptions show the most extreme form of differential effects (opposite signs at different levels of analyses), results for pity differ because the associations are of the same sign across levels, yet societal meta-perceptions of pity have predictive power over and above older people’s personal meta-perceptions. It is conceivable that older people have internalized the societal idea that old age is associated with passiveness, neediness, and frailty (cf. Levy, 2009) and, therefore, tend to behave accordingly triggering ageist behavioral responses in others, which in turn increase their PAD. However, beyond these subjective perceptions there is also strong evidence that pity has emergent properties when aggregated to the group level. This corroborates past research in which it has been argued that pity is a central emotion felt toward older people (e.g., Fiske et al., 2002). Our results indicate that this ambivalent emotion is indeed a powerful variable operating at both the individual- and societal-level which suggests that intervention schemes, targeting specifically pity perceptions, need to be directed at both older individuals as well as societies in order to counteract PAD.

Finally, competence and contempt were the only meta-perceptions that predicted age discrimination at the individual-level but not at the societal-level. This points to an important aspect regarding the social construction of the aging process. While contemporary aging theories argue that older people simply adopt societal views on old age across the life span (see, e.g., Levy, 2009), these findings suggest a more complex picture. The meta-perception of competence is usually related to perceptions of physical and cognitive declines which are frequently explained in terms of’inevitable’ biological outcomes of aging (cf. Levy, 2003). Hence, it might be that this specific notion of aging becomes highly self-relevant once individuals enter old age. Furthermore, this self-relevance might lead to self-fulfilling prophecies and therefore heighten the perception of age discrimination. This association does not occur at the societal-level because the self-relevance aspect means that it is an entirely psychological phenomenon that might increase older people’s sensitivity for age discrimination. A similar mechanism may apply to contempt, which includes feelings of disgust and possibly general anxieties due to the fact that aging can bring about illness and disability and eventually death. Again, this should become highly self-relevant for older people which in turn may make them more sensitive to any corresponding ageist behaviors. We are aware that more research is needed in order to shed light on these tentative speculations.

#### Beyond meta-perceptions: Social norms

We also examined social norms of intolerance of age prejudice as country-level predictors of older peoples’ PAD. The motivation to be unprejudiced has been studied extensively at the individual-level (Kunstman et al., 2013), yet our study is the first to show its predictive power as a variable characterizing the social climate on older people’s PAD. In countries in which people think that it is important to be unprejudiced toward other age groups, older people reported less instances of age discrimination. Moreover, this variable trumped all societal-level meta-perceptions in the prediction of age discrimination suggesting that implementing social norms promoting tolerance are an important factor to improve older people’s aging experience.

It has long been argued that a macro-level perspective can substantially augment psychological accounts of collective realities in which social forces operate in a larger societal context that contribute to individual behavior (Oishi & Graham, 2010). In the present article, we showed that the social climate is related to older people’s aging experience over and above personal and societal meta-perceptions. This has important implications for society given that the motivation to be unprejudiced as a social norm can be expressed and reinforced in institutions implicitly as well as explicitly, for example, in terms of choices of terms to refer to older people and their legal rights. Here we have shown that the social climate plausibly has significant consequences in terms of how older people are treated within a society as a whole, regardless of people’s personal attitude toward them (cf. Frymer, 2005). Thus one important avenue for policy interventions to tackle age discrimination is to introduce or increase awareness of social norms that actively inhibit prejudice toward other age groups, especially in some national contexts.

### Limitations

The ESS data were collected at the height of the global financial crisis during 2008 to 2010 raising the question of whether the findings are specific to this particular time period. Cross-national longitudinal data on this topic would be insightful into how these changes affect meta-perceptions, norms and perceived discrimination. However, the ESS Round 4 data remain the only cross-national data available, so at present we can do no more than speculate regarding period effects.

Our decision to focus on the aging experience of those over 70 years of age raises the question of whether the findings may generalize to the young−old age group. To gauge this possibility, we repeated the analyses in Model 1b (see Table 4) with individual-level meta-perceptions and covariates as predictors of PAD using a sample of respondents over 50 years of age (*N* = 25,814; *M*_*age*_ 64.63, *SD* = 10.00; 56.3% female). We found no difference in the results compared to the subsample with respondents over the age of 70.^8^ Hence, the results remain stable even when considering a more inclusive age range. This might be explained by the fact that age is a continuous variable (unlike gender and race).

Regarding societal meta-perceptions, it might also be that the young-old (60 to 69 years of age) do not share the outgroup perspective on old age. To assess whether this would make a difference, we correlated societal meta-perceptions based on those younger than 70 years of age with those younger than 60 years of age. The correlations show that they are almost identical ranging from (*r* = .993 for pity to *r* = .999 to social status). Hence, excluding the young−old would not make a difference in the data representing societal views on age.

We are aware that cross-sectional evidence constrains interpretation of cause and effect. Thus we relied on theoretically specified hypotheses that meta-perceptions of old age should predict older people’s PAD. We think it is seems reasonable to assume that societal meta-perceptions of individuals under the age of 70 are not primarily caused by older people’s PAD. Hence, the greater ambiguity concerns the association between older peoples’personal meta-perceptions and PAD. Here the opposite direction of effects is indeed conceivable: older people who perceive age discriminatory acts may develop certain meta-beliefs about their age group. Previous experimental studies support the hypothesized direction of effects from meta-perception to the experience of discrimination (Frey & Tropp, 2006; Vorauer, Hunter, Main, & Roy, 2000), but it is plausible that a more complex bidirectional causation exists (see also Vorauer & Kumhyr, 2001).

The evidence is also limited by the use of single item measures, in common with that from most major social surveys. Items in the ESS meet the highest methodological standards in survey research to ensure reliability and validity. They are pilot tested extensively for construct validity and are subjected to scrutiny, peer review and evaluation by the ESS Central Coordinating Team. In addition, the measures in this research had been used in numerous previous surveys, bolstering confidence that they are good indicators of ageist beliefs that can be meaningfully interpreted (for further reliability and validity analyses, see also Vauclair et al., 2010).

### Conclusion

Population aging poses a number of challenges for government budgets. Age discrimination constitutes an important psycho-social stressor which can increase the risk of ill-health (cf. Pascoe & Smart Richman, 2009; Vauclair et al., 2015a). Hence, understanding how personal and societal aging perceptions may bear on older people’s experience of age discrimination is an important step toward developing appropriate counter-ageism strategies. The present evidence suggests that interventions need to be directed at multiple levels and must target different elements of beliefs about older people as well as social norms about age prejudice. We hope that future research efforts will continue to develop a fuller understanding of a multilevel perspective on ageism.

## Supplementary Material

10.1037/pag0000125.supp

## Figures and Tables

**Table 1:** Overview of Hypotheses, Levels of Analyses, and Empirical Results

Hypothesis	Predictor	Level of analysis	Hypothesized association with older people’s experience of age discrimination	Result	Conclusion about hypothesis
1a	Social status of 70+	Individual	Negative	Negative	Confirmed
1b	Social status of 70+	Societal	Negative	Negative	Confirmed
2a	Warmth of 70+	Individual	Negative	Negative	Confirmed
2b	Warmth of 70+	Societal	Negative	Negative	Confirmed
3a	Competence of 70+	Individual	Negative	Negative	Confirmed
3b	Competence of 70+	Societal	Negative	*ns*	Rejected
4a	Pity toward 70+	Individual	Positive	Positive	Confirmed
4b	Pity toward 70+	Societal	Positive	Positive	Confirmed
5a	Envy toward 70+	Individual	Positive	Positive	Confirmed
5b	Envy toward 70+	Societal	Positive	Negative	Rejected
6a	Contempt toward 70+	Individual	Positive	Positive	Confirmed
6b	Contempt towards 70+	Societal	Positive	*ns*	Rejected
7	Social norms of intolerance of age prejudice	Societal	Negative	Negative	Confirmed
*Note.* 70+ = people aged 70 years and older.					

**Table 2 tbl2:** Descriptive Statistics of the National Samples Used in the Multilevel Regression Analyses

	Respondents over 70 years of age					Respondents under 69 years of age			
		Age		Female	Age discrimination more than once		Age		Female
Country	*N*	*M*	*SD*	%	%	*N*	*M*	*SD*	%
Belgium	236	77.38	5.88	57	38.56	1,524	41.67	15.12	50
Bulgaria	390	76.11	4.94	52	50.39	1,840	46.70	14.88	57
Croatia	214	75.62	4.58	57	34.43	1,239	42.42	15.00	57
Cyprus	130	74.95	4.46	45	48.46	1,085	41.20	15.16	50
Czech Republic	203	76.37	5.22	63	76.35	1,815	43.82	14.99	50
Denmark	215	77.41	6.21	52	30.14	1,395	44.92	15.16	50
Estonia	272	76.84	5.53	69	39.26	1,389	42.09	15.46	55
Finland	314	77.33	5.75	62	39.74	1,881	43.07	15.40	49
France	332	77.97	5.64	60	38.72	1,741	43.06	14.69	54
Germany	374	76.02	4.93	52	45.99	2,351	44.65	14.60	47
Greece	186	75.69	5.01	52	54.89	1,884	42.01	14.28	55
Hungary	242	77.48	5.60	60	48.76	1,302	42.26	15.20	53
Ireland	244	76.85	5.18	55	25.00	1,509	42.89	14.53	54
Israel	323	77.50	5.63	53	36.60	2,131	40.56	15.34	54
Latvia	307	76.05	4.87	74	44.59	1,673	43.23	15.39	60
Netherlands	269	77.36	5.58	58	34.33	1,509	44.31	14.20	53
Norway	163	77.49	5.95	52	32.10	1,385	42.02	14.82	48
Poland	204	76.65	4.76	58	39.80	1,415	40.03	15.46	52
Portugal	609	77.22	5.27	66	32.13	1,757	44.26	15.72	59
Romania	256	75.66	4.48	54	59.60	1,859	42.01	14.67	55
Russian Federation	388	76.31	5.20	71	66.49	2,120	41.88	15.44	59
Slovakia	255	76.06	5.16	80	68.63	1,543	45.79	14.44	59
Slovenia	187	76.67	5.38	63	30.65	1,099	41.43	15.26	52
Spain	410	77.71	5.77	56	41.08	2,162	40.97	14.67	52
Sweden	283	77.60	5.51	57	24.10	1,547	42.11	15.45	49
Switzerland	292	77.66	5.59	60	36.30	1,527	43.03	14.22	54
Turkey	143	76.18	5.74	50	38.46	2,252	37.28	14.02	54
Ukraine	301	76.33	5.27	70	63.21	1,544	43.47	15.34	61
United Kingdom	381	78.08	6.20	53	34.04	1,943	43.48	14.45	54
Total	8,123	60	76.90	5.43	43.20	48,421	42.60	15.10	54

**Table 3 tbl3:** Multilevel Regression Models With Only Meta-Perceptions as Predictors at the Individual- and Societal-Levels

Predictor	Hypotheses and tests of personal and societal meta-perceptions																	
	H1a^a^	H1b^b^	Both^c^	H2a	H2b	Both	H3a	H3b	Both	H4a	H4b	Both	H5a	H5b	Both	H6a	H6b	Both
Individual-level																		
Status	−.03***		−.03***															
Friendliness				−.10***		−.10***												
Competence							−.04^c^		−.05***									
Pity										.09***		.09***						
Envy													.06***		.06***			
Contempt																.17***		.17***
Societal-level																		
Status		−.13**	−.11*															
Friendliness					−.68**	−**.60**												
Competence								−.04	**.02**									
Pity											.75***	.67***						
Envy														−.44**	−.55***			
Contempt																	.25	**.13**
*Note.* Values in bold denote results that lend more support to psychological phenomena. Analyses were conducted by using the design weight (as provided by the European Social Survey [ESS; European Social Survey Round 4 Data, 2008]) and individual-level covariates to adjust for a possible sampling bias and compositional effects, respectively. The results were virtually identical without individual-level covariates.																		
^a^ Hypotheses with an “a” suffix are group-mean centered. ^b^ Hypotheses with a “b” suffix are grand-mean centered. ^c^ *Both* refers to simultaneous tests of individual- and societal-level meta-perceptions. All predictors are grand-mean centered.																		
* *p* < .05. ** *p* < .01. *** *p* < .001.																		

**Table 4 tbl4:** Full Multilevel Regression Models Predicting Older People’s Perceived Age Discrimination

	Model 0	Model 1a	Model 1b	Model 2a	Model 2b	Model 3
	Intercept					
Predictor	.527***	.568***	.571***	.597***	.598***	.599***
Individual-level predictors						
Female		.034	.038	.039	.038	.038
Born in country		.048	.041	.043	.037	.03
Age		.002	.004	.004	.004	.004
Education		.004	.006	.007**	.007*	.007*
Subjective poverty		.104***	.107***	.110***	.108***	.106***
Subjective ill-health		.071***	.064***	.064***	.063***	.062***
Life satisfaction		−.041***	−.037***	−.036***	−.036***	−.036***
Seriousness ageism		.118***	.110***	.111***	.111***	.109***
Generalized trust		−.026***	−.022***	−.022***	−.021***	−.021***
Status			−.016**	−.015**	−.015**	−.016**
Positive age stereotype^a^			−.075***	−.075***	−.075***	−.075***
Pity			.031**	.032**	.033**	.031**
Contempt			.137***	.138***	.138***	.138***
Envy			.027**	.025*	.025*	.027**
Country-level predictors^b^						
HDI				−.744		
Gini coefficient				−.002		
Status						.065
Friendliness						.031
Pity						.197
Envy						−.12
Norm of intolerance of age prejudice					−.125**	−.139**
Variance components						
Individual-level	.666	.613	.553	.553	.553	.553
Country-level	.063***	.076***	.077***	.022***	.016***	.012***
Model fit statistics						
Deviance	19,687	14,982	12,801	12,768	12,761	12,753
*df*	3	12	17	19	18	22
Explained variance						
Individual-level (%)		7.97	16.97			
Country-level (%)				65.08	74.6	80.95
*Note.* Individual-level predictors in Model 1a and 1b are group-mean centered and in Model 2a, 2b, and 3 grand-mean centered. Analyses were conducted by using the design weight (as provided by the European Social Survey [ESS; European Social Survey Round 4 Data, 2008]) to adjust for a possible sampling bias. HDI = Human Development Index.						
^a^ This is an index composed of friendliness and warmth ratings because of the high inter-item correlation at the individual-level. ^b^ Only country-level variables that emerged as significant predictors in previous analyses are included.						
* *p* < .05. ** *p* < .01. *** *p* < .001.						

## Footnotes

1 Recent theorizing on the perception of different social groups also suggests a mediation model that includes these different elements of meta-perceptions (Fiske et al., 2002). Unfortunately, such a model is too complex to be tested at multiple levels due to the relatively low country-level sample size.

2 The interaction between warmth and competence (see Cuddy et al., 2007) turned out to be nonsignificant and was, therefore, not taken into account in our analyses.

3 This variable assesses the extent to which older people are sensitive to the issue of age discrimination as a societal problem (Abrams et al., 2011). Their responses may be informed by their own perceived age discrimination, but also by other societal factors such as media and policy directives. The results were virtually the same with and without this covariate.

4 The ESS also includes a question on admiration. We included this variable in our initial analyses and found that it was the only metaperception that was not significantly associated with PAD at any level of analysis. Due to space limitations we dropped it from the article.

5 Please see http://appsso.eurostat.ec.europa.eu/nui/show.do?dataset=ilc_di12&lang=en

6 Please see http://www.wider.unu.edu/research/Database/

7 We also examined whether the results change when examining the “treated badly” and “lack of respect” items as separate criterion variables. As one of the reviewers suggested, it is conceivable that pity would predict patronizing/benevolent prejudice experiences (i.e., lack of respect), whereas contempt and envy would predict more hostile experiences (i.e., bad treatment). However, we did not find evidence for this. The pattern of results was very similar for these two items and matched the ones reported in Table 3; bad treatment item: pity_Level 1_: *B* = 0.075, *p* < .001 and pity_Level 2_: *B* = 0.583, *p* < .001; envy_Level 1_: *B* = 0.050, *p* < .001, and envy_Level 2_: *B* = −0.431, *p* < .01; contempt_Level 1_: *B* = 0.161, *p* < .001 and contempt_Level 2_: *B* = 0.125, *p* = .594; lack of respect item: pity_Level 1_: *B* = 0.099, *p* < .001, and pity_Level 2_: *B* = 0.617, *p* < .001; envy_Level 1_: *B* = 0.059, *p* < .001, and envy_Level 2_: *B* = −0.525, *p* < .001; contempt_Level 1_: *B* = 0.198, *p* < .001, and contempt_Level 2_: *B* = 0.145, *p* = .579.

8 Status: *B* = −0.017, *p* < .001; age stereotypes: *B* = 0.041, *p* < .001; pity: *B* = 0.022, *p* < .001; contempt: *B* = 0.126, *p* < .001; envy: *B* = 0.045, *p* < .001.
