# Supplementary material for: What Do Older People Think That Others Think of Them, and Does It Matter? The Role of Meta-Perceptions and Social Norms in the Prediction of Perceived Age Discrimination
Source: Psychol Aging. 2016 Nov;31(7):699–710. doi: 10.1037/pag0000125 (PMC5104248; doi:10.1037/pag0000125)
Supplement: Supplementary file 1 [file Supplementary.docx]

Supplementary Table 1

*Multilevel regression models predicting older people´s perceived age discrimination without individual-level covariates*

|  |  | Models | | | | |
| --- | --- | --- | --- | --- | --- | --- |
|  |  | 0 | 1 | 2a | 2b | 3 |
|  | Intercept | 0.527^c^ | 0.537^c^ | 0.549^c^ | 0.550^c^ | 0.553^c^ |
|  |  |  |  |  |  |  |
| Individual-Level Predictors | | |  |  |  |  |
|  | Status |  | -0.031^c^ | -0.031c | -0.031c | -0.016^b^ |
|  | Positive Age Stereotype^1^ | | -0.089^c^ | -0.089^c^ | -0.089^c^ | -0.075^c^ |
|  | Pity |  | 0.035^c^ | 0.037c | 0.037c | 0.031^b^ |
|  | Contempt | | 0.153^c^ | 0.155^c^ | 0.155^c^ | 0.138^c^ |
|  | Envy |  | 0.025^a^ | 0.022^a^ | 0.022^a^ | 0.027^b^ |
|  |  |  |  |  |  |  |
| Country-Level Predictors^2^ | | |  |  |  |  |
|  | HDI |  |  | -2.443^b^ |  |  |
|  | GINI |  |  | -0.002 |  |  |
|  |  |  |  |  |  |  |
|  | Status |  |  |  |  | 0.044 |
|  | Friendliness | |  |  |  | -0.146 |
|  | Pity |  |  |  |  | 0.265^a^ |
|  | Envy |  |  |  |  | -0.200^a^ |
|  | Norm of Intolerance of Age Prejudice |  |  |  | -0.232^c^ | -0.177^c^ |
| Variance Components | |  |  |  |  |  |
|  | Individual-Level | 0.666 | 0.59 | 0.591 | 0.591 | 0.591 |
|  | Country-Level | 0.063^c^ | 0.065^c^ | 0.029^c^ | 0.020^c^ | 0.010^c^ |
| Model Fit Statistics | |  |  |  |  |  |
|  | Deviance | 19687 | 15996 | 15975 | 15965 | 15949 |
|  | df | 3 | 8 | 10 | 9 | 13 |
| Explained Variance | |  |  |  |  |  |
|  | Individual-Level (%) | | 11.41 |  |  |  |
|  | Country-Level (%) | |  | 53.97 | 68.25 | 84.13 |

*Note.* ^a^*p* < .05; ^b^*p* < .01; ^c^*p* < .001 (two-tailed). ^1^ This is an index composed of friendliness and warmth ratings because of the high inter-item correlation at the individual-level. ^2^ Only country-level variables that emerged as significant predictors in previous analyses are included (see Table 3). Individual-level predictors in Model 1a are group-mean centered and in Model 2a, 2b and 3 grand-mean centered. Analyses were conducted by using the design weight (as provided by the ESS) to adjust for a possible sampling bias.
